# Geographical Heterogeneity in Antimalarial Resistance Markers Revealed by Genomic Surveillance in Angola, 2023

**DOI:** 10.1101/2025.04.08.25325242

**Published:** 2025-04-10

**Authors:** Maria Florinda João, Andrés Aranda-Díaz, Faith De Amaral, Takalani I. Makhanthisa, Sonja B. Lauterbach, Mukosha Chisenga, Brighton Mangena, Paulo Maquina, Isobel Routledge, Chadwick Sikaala, John Chimumbwa, Domingos Jandondo, José Franco Martins, Jaishree Raman, Jennifer L. Smith, Pedro Rafael Dimbu

**Affiliations:** 1 Programa Nacional do Controlo da Malária, Luanda, Angola; 2 EPPIcenter Research Program, Department of Medicine, University of California, San Francisco, United States of America; 3 Malaria Elimination Initiative, Global Health Group, University of California, San Francisco, United States of America; 4 ISGlobal Barcelona, Spain; 5 Laboratory for Antimalarial Resistance Monitoring and Malaria Operational Research, Centre of Emerging Zoonotic and Parasitic Diseases, National Institute for Communicable Diseases, Johannesburg, South Africa; 6 SADC Malaria Elimination Eight Secretariat, Windhoek, Namibia; 7 Instituto Nacional de Investigação em Saúde, Luanda, Angola; 8 Wits Research Institute for Malaria, University of Witwatersrand, Johannesburg, South Africa; 9 University of Pretoria Institute for Sustainable Malaria Control, University of Pretoria, Pretoria, South Africa; 10 Department of Epidemiology & Biostatistics, University of California, San Francisco, United States of America

## Abstract

*Plasmodium falciparum* malaria remains a leading cause of mortality in Angola, with emerging antimalarial resistance threatening treatment and prevention strategies. Efficacy of artemether-lumefantrine, one of the country’s preferred malaria treatments, has been reported below 90% in two provinces, underscoring the need for routine resistance surveillance and efficacy monitoring to guide policy decisions.

Between March and July 2023, dried blood spots and demographic data were collected from *P*. *falciparum*-positive participants at 16 health facilities across 8 provinces. Multiplexed amplicon deep sequencing was used to characterize single nucleotide polymorphisms in 12 genes linked with resistance, estimate allele frequencies, and detect co-infecting non-falciparum Plasmodium species.

Sequence data from 817 samples revealed significant geographic variation in resistance markers. In the southeast, artemisinin partial resistance markers (*k13* P574L, P441L), were detected at very low prevalence (<0.1%), while the quintuple *dhps*/*dhfr* haplotype, linked to sulfadoxine-pyrimethamine (SP) resistance, was very prevalent (>40% of samples). In the northwest, the sextuple *dhps*/*dhfr* haplotype, a marker of higher SP resistance, was most prevalent in Zaire (14.2%). The *crt* CVIET haplotype, associated with chloroquine resistance, had a national prevalence of 15.9%, detected in over 48% of samples from Zaire and Uíge. The *mdr1* N86 genotype, linked to reduced lumefantrine susceptibility, was widespread, detected in 99.3% of samples. Co-infections of *P*. *falciparum* and non-falciparum species were rare with no clear geographic distribution. No *P*. *vivax* co-infections were detected.

These findings highlight the need for continued monitoring to safeguard treatment efficacy, reinforcing the importance of molecular surveillance in malaria control strategies.

## Introduction

Angola is the seventh-most malaria burdened country, globally, reporting 10,488,709 cases and 10,089 deaths in 2023 according to unpublished data from the Ministry of Health’s DHIS2 system.^1^ Malaria control relies on vector control using long-lasting insecticide-treated nets, indoor residual spraying, fumigation, and larviciding, together with case management strategies that ensure rapid and effective diagnosis and treatment of malaria infections.^2^ Preventive treatments for at-risk populations, including seasonal chemoprevention and intermittent preventive treatment in pregnancy (IPTp), are complemented by efforts to promote community engagement and education of local leaders. However, operational and biological challenges, particularly the emergence of resistance to antimalarials, threaten malaria control efforts.^3^

Chloroquine was used to treat uncomplicated malaria in Angola until the early 2000s, when confirmed chloroquine resistance prompted a transition to amodiaquine or sulfadoxine-pyrimethamine (SP).^4,5^ Increasing levels of SP resistance led to the adoption of artemisinin-based combination therapies (ACTs), as the World Health Organization (WHO) recommended first line treatment in 2006, with implementation completed by 2008.^4,6^ Artemisinin partial resistance (ART-R), characterized by delayed parasite clearance, is widespread in Southeast Asia and has recently emerged in East and the Horn of Africa.^7–10^

Four out of the five therapeutic efficacy studies conducted in Angola since 2013 have reported artemether-lumefantrine (AL) efficacies below 90%, the WHO threshold at which further investigation and potential revisions to national treatment guidelines are recommended.^11–15^ These reports of reduced AL efficacy are localized primarily to the northern province of Zaire. The other ACTs used in-country, namely artesunate-amodiaquine (ASAQ), dihydroartemisinin-piperaquine (DHAP), and artesunate-pyronaridine (ASPY), have remained effective.^11–15^ No studies evaluating the chemopreventive efficacy of SP have been conducted in Angola. However, reports from studies elsewhere suggest that SP-based IPTp continues to provide clinical benefits, even in the presence of resistant strains.^16–18^

Molecular surveillance of mutations associated with antimalarial resistance complements therapeutic efficacy and chemopreventive efficacy studies, serving as an early warning mechanism and guiding efficacy assessments.^19^ ART-R is associated with single nucleotide polymorphisms (SNPs) in the propeller domain of the Kelch13 (K13) protein, encoded by the *k13* gene.^20^ These SNPs are classified based on the available clinical or laboratory evidence: validated markers require both types of evidence, while candidate markers are supported by only one.^21^ Over the past decade, validated markers C469Y, R561H, R622I, and A675V, along with the candidate markers P441L and C469F, have been increasingly reported in Ethiopia, Eritrea, Tanzania, Uganda, and Rwanda.^9,10,22–25^

Chloroquine resistance is associated with mutations in the chloroquine resistance transporter (*crt*) gene, particularly the K76T mutation within the 72–76 CVIET haplotype, which was dominant in Africa.^26–28^ Chloroquine resistance is further modulated by other mutations in *crt* and in the multidrug resistance (*mdr1*) gene. Mutations in *crt* and *mdr1* genes are also associated with resistance or reduced susceptibility to piperaquine, amodiaquine, and lumefantrine.^29,30^ Copy number variations (CNV) in the *plasmepsin 2* and *3* genes are associated with piperaquine resistance, while duplications in *mdr1 gene* are associated with mefloquine resistance. Resistance to SP is driven by the accumulation of SNPs in the dihydropteroate synthase (*dhps*) and dihydrofolate reductase (*dhfr*) genes.^31^

Prior studies in Angola over the past decade have reported a very low prevalence of *k13* mutations, with no validated or candidate ART-R markers detected.^32–36^ The chloroquine resistance *crt* K76T marker, and the *mdr1* N86 genotype, linked to reduced lumefantrine susceptibility were frequently identified, but mutations in the *dhps* and *dhfr* genes associated with high SP resistance have remained low.^14,32,34,36^ The rapid emergence of resistance markers in East and the Horn of Africa underscores the need for routine, geographically representative molecular surveillance to inform timely policies.

To provide a geographically comprehensive and up-to-date overview of antimalarial drug resistance markers in Angola, the Genomics for Malaria in the Elimination 8 (GenE8) initiative - a regional effort aimed at strengthening malaria genomic surveillance in five Southern African countries (Angola, Eswatini, Namibia, South Africa, and Zambia) - conducted molecular malaria surveillance using targeted amplicon deep sequencing across 8 provinces of Angola. The prevalence of molecular markers associated with resistance to the most used antimalarials, based on malaria-positive samples collected between April and July 2023 is presented here.

## Materials and Methods

### Study Site

**Figure 1:**
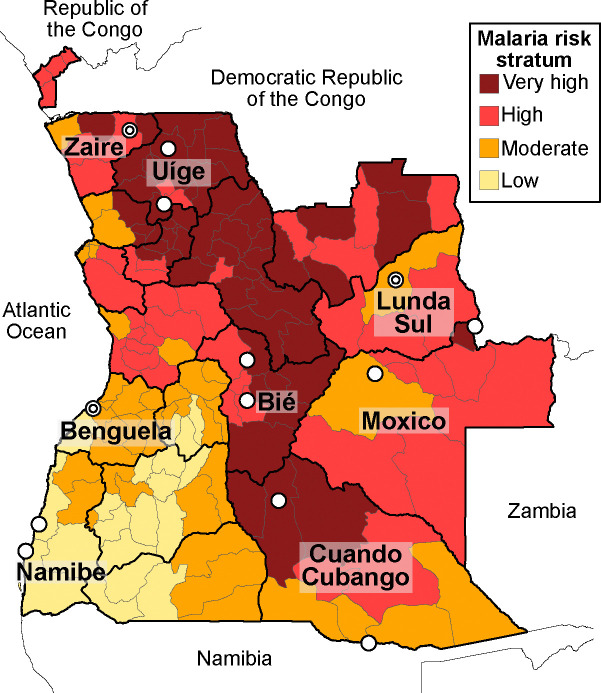
Angola malaria risk stratifications. ^2^ Risk strata are shown in different colors (see Study Site section for definitions). Health facilities where samples were collected are indicated with circles, with overlapping facilities represented by two concentric circles.

Angola comprises 18 provinces and share borders with the Democratic Republic of the Congo, Zambia, and Namibia (Figure 1). Malaria prevalence, based on rapid diagnostic tests (RDTs), is 17% among children aged 6 to 59 months.^37,38^ Since transmission intensity varies significantly across the country and within provinces, malaria transmission is stratified at the municipal level (Figure 1) into 4 categories: very high (incidence >1,000 cases per 1,000 inhabitants, prevalence >50%), high (incidence 500–1,000/1,000, prevalence between 30–50%), moderate (incidence 300–500/1,000 inhabitants, prevalence 10–30%), and low (incidence <300/1,000, prevalence <10%).^39^ Transmission occurs year-round, with peaks between March and May. The predominant parasite species is *Plasmodium falciparum*, although *P*. *malariae*, *P*. *ovale*, and *P*. *vivax* are occasionally reported.^40,41^ The primary malaria vectors include *Anopheles gambiae* s.l., *An*. *arabiensis*, and *An*. *funestus* s.l.^39^ This study was conducted in 8 provinces (Figure 1) where 100% of the population, estimated at 10.5 million (30.9% of the total population), is at risk of contracting malaria. The healthcare system in these 8 provinces comprises 897 health facilities that serve as malaria diagnosis and treatment centers. Diagnosis is conducted at the point of care using *P*. *falciparum* histidine-rich protein 2 (HRP2)-based RDTs or microscopy. All patients with uncomplicated malaria receive one of three first-line ACTs (AL, ASAQ, or DHAP). Severe malaria cases are treated with intravenous artesunate, intramuscular artemether, or injectable quinine, followed by an ACT. SP is used as IPTp in all health facilities with antenatal care services.

### Study Design, and Field Procedures

This cross-sectional, health facility-based study was conducted as part of the Regional GenE8 (ReGenE8) project and was integrated into Angola’s national surveillance system. Eight provinces, with two health facilities each were selected ensuring geographic and transmission-strata representativeness.

Zaire, Lunda Sul, and Benguela provinces each host two therapeutic efficacy study sites which were included in this study. The remaining 10 health facilities were selected from the remaining five provinces (Bié, Cuando Cubango, Moxico, Namibe and Uíge), ensuring representation across all four transmission strata, while taking accessibility and patient flow into consideration. The study aimed to successfully sequence at least 50 RDT-positive samples from each of 16 health facilities, To achieve this, 200 RDT-positive patients were targeted for dried blood spot (DBS) sample and demographic data collection. The total sequenced target sample size (n=800) was designed to: a) estimate a 5% prevalence of a specific molecular marker with 1.85% precision at 95% confidence, and b) achieve 90% power to detect a ≥2% prevalence if the true prevalence is 4.16% using a one-sided binomial test (α=0.05, design effect (Deff)=1.5). The targeted sample size was inflated to 200 RDT-positive patients per facility to ensure high-quality sequencing of the minimum sample size and retain additional samples in a regional biobank for follow-up investigations. Study activities took place from March to July 2023, during the malaria transmission season, with recruitment staggered across health facilities. All symptomatic patients over two years old who tested positive for *P*. *falciparum* malaria by HRP2-based RDT (SD Bioline P.f./P.v.) were invited to participate in the study. Patients with a history of fever in the previous two weeks or severe malaria were excluded. In most facilities, sample and data collection targets were met within two weeks. However, the collection period was extended to 3 months in Hospital Geral de Benguela (Benguela province), and Hospital Municipal do Calai (bordering Namibia in Cuando Cubango province) due to low case numbers.

Prior to study enrollment, written informed consent was obtained as described in the Ethical Considerations section. Recruited participants were interviewed at the point of care using paper forms to collect demographic data, clinical symptoms, travel history, and occupation. Finger-prick DBS samples were collected on filter paper strips (Cytiva Whatmann 3MM Chr), labeled with unique barcodes linking survey data, air-dried, and stored individually in sealed bags with desiccant. Labelled samples were sent to the National Institute of Health Research in Luanda, Angola, before being shipped to the regional sequencing hub at the National Institute for Communicable Diseases in South Africa. Survey forms were stored at the National Malaria Control Program, where the demographic data were digitized using CommCare software on encrypted Android tablets.

### Sample Processing and Genomic Assay

DNA was extracted from at least 90 samples per health facility (one 6 mm DBS disc per sample) using the Chelex-Tween 20 method.^42^ Parasite densities were estimated using the varATS qPCR assay.^43^ A minimum of 70 *P*. *falciparum*-positive samples per health facility with >1,000 parasites/μL of blood were randomly selected for sequencing. If this target could not be achieved, samples with parasitemia as low as 10 parasites/μL of blood were included.

Amplicon sequencing libraries were prepared using the D1.1, R1.2, and R2.1 pools of the MAD4HatTeR targeted amplicon sequencing panel and sequenced on a NextSeq 2000 instrument using 150-cycle paired-end reads.^44^ A custom bioinformatics pipeline was used to infer alleles at 241 targets.^44^ This report includes sequences from the following genes: *k13*, *crt*, *mdr1*, multidrug resistance 2 (*mdr2)*, *dhps*, *dhfr*, *coronin*, apicoplast ribosomal protein S10 (*arps10*), putative phosphoinositide-binding protein (*pib7*), ferrodoxin (*fd*), exonuclease (*exo)*, and PF3D7_1322700. Lactate dehydrogenase (*ldh*) gene target sequences in *P*. *falciparum*, *P*. *vivax*, *P*. *malariae*, *P*. *ovale* and *P*. *knowlesi* were used to identify the presence of *Plasmodium* species in the samples. SNPs in *ldh* targets were used to distinguish *P*. *ovale curtisi* from *P*. *ovale wallikeri*. Microhaplotypes are reported for SNPs within the same amplicon. Variations to the original protocol and bioinformatics pipeline are described in Eloff, Aranda-Díaz et al, 2025.^45^

## Data Cleaning, Analysis, and Visualization

Data cleaning, analysis, and visualization were conducted in R (version 4.3.1). Samples with <10,000 reads, targets with <50 reads, and SNPs with within-sample allele frequency (WSAF) <0.01 or with ≤10 reads were excluded from the analysis. SNPs observed in only one sample with WSAF <1, and SNPs observed in multiple samples but exclusively in a single sequencing run, were also excluded. Library preparation batches with high contamination in negative or positive controls were also excluded.

Non-*falciparum* targets were excluded from sequencing depth summaries. Otherwise, all resulting data were analyzed, including samples with partial SNP coverage, leading to variable total sample numbers for each assessed SNP. Samples were considered successfully sequenced if a genotype was obtained for all 59 SNPs evaluated in *k13 gene*. Multilocus haplotypes for polymorphisms in 5 independent targets in *dhps* and *dhfr* genes (*dhps* 436, 437, 540, 581, and 613; and *dhfr* 51, 59, 108, and 164), 3 independent targets in the *mdr1* gene (amino acids 86, 184 and 1246), and 3 independent targets in the *crt* gene (*crt* 72–76, 220, 356) were inferred for each sample by combining all individual haplotypes. Haplotypes were inferred for samples with either unmixed genotypes at all targets or with a single target showing a mixed genotype, in which case the sample was assumed to carry the two haplotypes accounting for the observed mixture. Haplotype classification for *dhps* (wild type, <2 mutations, double or triple), *dhfr* (wild type, <3 mutations, triple or quadruple) and *dhps*/*dhfr* (wild type, <5 mutations, quintuple or sextuple) are detailed in Table S1. Samples with mixed genotypes in more than two amplicons were considered indeterminate.

To assess data patterns and identify potential issues with sampling and library preparation, sample similarity was estimated using the root mean square error of WSAF for highly diverse loci (pool D1.1 targets), followed by Density-Based Spatial Clustering of Applications with Noise (DBSCAN) with ε=0.2. Samples with clonality >2 and nearly identical WSAF across all loci, suggesting contamination or sample duplication, were excluded from the analysis.

### Statistical Analysis

Descriptive statistics for gender, age group (<5, 5–14, 15–24, 25–39, 40–59, >59 years), occupation (including the self-defined occupation category ‘minor’), overnight travel in the past two months, and country of residence were calculated. To facilitate statistical analysis, some variables were reclassified: occupations in agriculture, herding, and fishing were combined into a single occupational category (agricultural workers); individuals aged 25 years or older were grouped into a single category; and travel reports (within two months before sample collection) were categorized as international or domestic. Differences between successfully sequenced samples and qPCR-positive samples, as well as between provinces, were assessed using Chi-square tests.

The proportion of samples carrying a mutation or haplotype was calculated as the number of mixed and pure mutant genotypes divided by the total number of genotyped infections. Proportions of the *dhps* and *dhfr* haplotypes were determined using the haplotype with the most mutations observed in each sample (Table S1). The prevalence of infections carrying a genotype at the national level was adjusted considering clustered sampling, and survey weights were applied to adjust for unequal sampling probabilities across provinces, health facilities, and participants using the R package *survey* (v.4.4.2). The *beta* method was used to obtain 95% confidence intervals.

Univariate logistic regression was performed to assess associations between the presence of markers of interest (binary outcome) and key categorical variables, including age group, gender, and occupation. Multivariable logistic regression was subsequently conducted using generalized linear mixed models, incorporating a random intercept for each health facility and fixed effects for all other variables. Due to the co-occurrence between certain occupational categories and age groups (e.g. agricultural workers predominantly in older groups), separate mixed-effects models were fitted: one including gender and age, and another including gender and occupation. Analyses were restricted to mutations and variables with a sufficient sample size to achieve model convergence.

The complexity of infection (COI) for each sample and population allele frequencies were jointly estimated for each municipality and province using MOIRE (v3.4.0), which implements a Markov chain Monte Carlo-based approach to jointly infer sample COI and within host relatedness, and population allele frequencies, using polyallelic genomic data.^46^ By modeling the genetic data and accounting for experimental errors, MOIRE provides probabilistic estimates. Microhaplotypes in all highly diverse *P. falciparum* targets (primer pool D1.1), as well as SNPs or micro-haplotypes in drug resistance targets of interest, were used to infer COI and allele frequencies. The percentage of polyclonal infections was estimated as the mean of individual probabilities of polyclonality.

Non-*falciparum* species were considered present if the combined reads across all *ldh* targets exceeded 100, with each species contributing more than 1% of the total *ldh* target reads.

### Data availability

A dataset containing all individual genotypes, COI and probabilities of polyclonality is available online (DOI:10.5281/zenodo.15126994).

### Ethical Considerations

The study received ethical approval from the Angolan Ministry of Health and the National Institute of Health Research. Written informed consent was obtained from all patients aged 18 years or older and from parents/guardians of children aged 2 to 11 years. For patients aged 12 to 18 years, verbal assent was obtained in addition to parental/guardian consent. Signed consent and survey forms were sent to the National Malaria Control Program, where they were stored in a secure, restricted-access location, with copies of the consent forms provided to participants or their parents/guardians. All personal identifiers were removed from survey data before storage in a password-protected cloud.

## Results

DNA was extracted from 1,656 samples, with sequencing libraries prepared for 1,024 (61.8%). Due to potential cross-contamination 23 sequenced samples were excluded from further analyses. At least one SNP genotype was obtained for 94.5% (946/1,001) of the samples from the 16 health facilities, and 817 (81.6%) were successfully sequenced (i.e. genotyped for all 59 *k13* SNPs). Sequencing success rates varied across provinces (Table 1), with Zaire having the highest number of successfully sequenced samples and Namibe the lowest. Due to low sample size at Zona Sul health facility in Namibe, this site was excluded from certain analyses.

The median sequencing depth per sample was 693,580 reads (interquartile range (IQR): 185,191–1,497,920), with a median depth per target of 1,342 reads (IQR: 266–4,362). A median of 223 out of 238 targets (IQR: 204–226) had more than 100 reads. SNPs with the lowest genotyping success rates included *crt* 145, *crt* 218/220, and *mdr1* 86 (genotyped in 751, 284, and 755 samples, respectively). More than two-thirds of the analyzed samples (68.4%) were polyclonal, with a population mean COI of 3.05 (95% confidence interval (CI): 3.00–3.12, N=946). Mixed genotypes in at least one drug resistance marker were detected in 57% of samples. Co-infecting non-*falciparum* species - *P. malariae*, *P. ovale curtisi*, and *P. ovale wallikeri* - were rare, collectively detected in 5.8% of samples (Table S2). No samples containing *P. vivax* were identified.

No significant differences in province, gender, or travel history were observed between individuals with qPCR-positive samples and those with successful sequenced samples (Table 1, Table S3). However, younger age groups and members of specific occupational categories (‘students’ and ‘minors’), were overrepresented among successfully sequenced samples compared to qPCR-positive samples. The majority of sequenced samples came from students (41%) or minors (36%), with only 15% of participants over 24 years old. Gender distribution was balanced (53% females), and all participants were Angolan. Travel was reported by 6.9% of participants, with just one case of international travel recorded.

### Mutations in the crt gene

**Figure 2.**
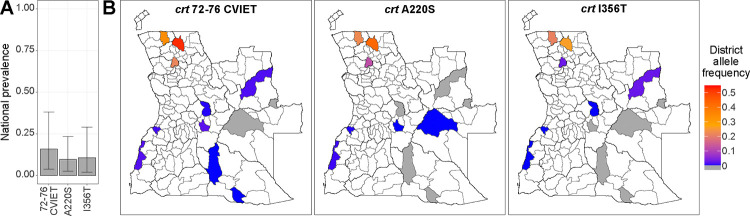
Mutations detected in the *crt* gene. **A**. National adjusted prevalence of infections carrying a *crt* genotype. **B**. Estimated allele frequencies at the municipal level for the detected *crt* genotypes.

Six non-synonymous mutations were detected in the *crt gene* (Table 2). The chloroquine-resistant *crt* 72–76 CVIET microhaplotype had an adjusted national prevalence of 15.9% (CI 3.9–38.0%, N=946, Deff=34.7, intraclass correlation coefficient (ICC)= 0.58, Figure 2A, Table S4) and was most abundant in the provinces of Uíge (58.9%, N=124) and Zaire (48.5%, N=134, Figure 2B, Table S5–6), where its allele frequency exceeded 21% (Table S7). In contrast, its abundance was low in the southeastern provinces of Cuando Cubango and Moxico (<2%, N=114 and 116). Similar adjusted national prevalences were observed for the *crt* A220S (9.6%, CI 2.5–23.4%, N=283, Deff=5.7, ICC=0.28) and I356T (10.6%, CI 2.1–29.0%, N=889, Deff=26.8, ICC=0.47, Figure 2A) mutations, which were also more frequent in the northern provinces (Figure 2B, Table S5–7). The *crt* A220S was mostly but not exclusively found in samples with the CVIET microhaplotype and *crt* I356T (Table S8). The *crt* I356T was always found in a haplotype with the CVIET microhaplotype and *crt* A220S.

Participants older than 24 years old had significantly higher odds of carrying the CVIET microhaplotype (odds ratio (OR): 2.66; CI: 1.31–5.41; p=0.007, Table S9–11) and the *crt* I356T mutation (OR: 2.36; CI: 1.04–5.34; p=0.04) compared to children under 5 years. Similarly, agricultural workers were significantly more likely to carry the CVIET microhaplotype (OR: 2.65; CI: 1.26–5.58; p=0.01) and the *crt* I356T mutation (OR: 2.45; CI: 1.05–5.72; p=0.038) compared to minors. No significant associations were observed for gender, and other occupational groups. Of the 154 samples carrying the CVIET microhaplotype, only four belonged to participants who reported domestic travel, and in two cases, travel occurred within the same district where they were sampled. Sample size was insufficient to assess associations between the *crt* A220S mutation with demographic factors.

### Mutations in the dhps and dhfr genotypes

**Figure 3.**
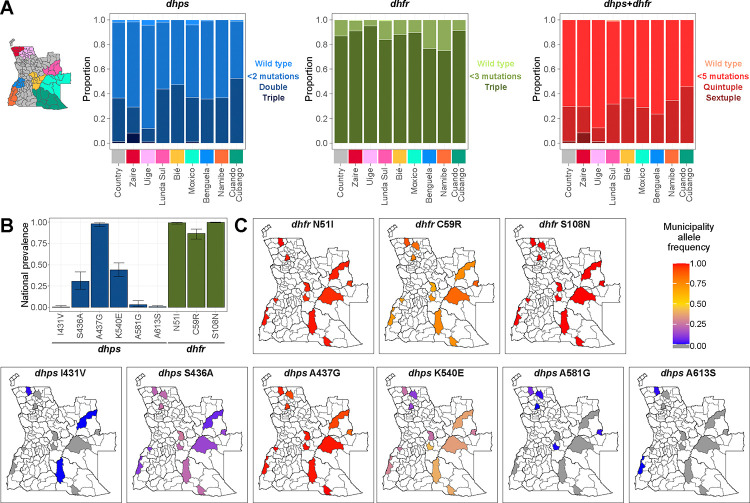
Mutations detected in the *dhps* and *dhfr genes*. **A**. Proportion of samples carrying a haplotype for *dhps*, *dhfr*, or *dhps+dhfr* at the provincial level, excluding undetermined samples. **B**. National adjusted prevalence of infections carrying a genotype in the *dhps* or *dhfr* gene. **C**. Estimated allele frequencies at the municipal level for the same markers.

A *dhps* haplotype could be inferred for 95.5% (857 of 897) of the samples with genotypes available at all four *dhps* SNPs, and a *dhfr* haplotype was inferred for all 918 samples genotyped for all three *dhfr* SNPs (Table S12–13). The *dhps* double mutant haplotype (S436A or A437G, plus K540E), associated with SP resistance, was present in 41.7% of the samples, while 2.7% carried the *dhps* triple mutant haplotype (A581G or A613S in addition to the double mutant haplotype), linked to higher SP resistance levels. The *dhps* triple mutant haplotype was particularly prevalent in northern Zaire province (Figure 3A). The *dhfr* triple mutant haplotype, linked to pyrimethamine resistance, was found in 89.9% of samples. Due to polyclonality and the presence of mixed genotypes, a full *dhps*/*dhfr* haplotype could be inferred for only 727 of 896 samples (81.1%) with complete genotypes for both genes (Table S14). Among these, 29.2% carried the quintuple mutant haplotype (*dhps* double mutant plus *dhfr* triple mutant), and 2.34% carried the sextuple mutant haplotype (*dhps* triple mutant plus *dhfr* triple mutant, Figure 3A).

The *dhps* A437G mutation had an adjusted national prevalence of 98.0% (CI 95.1–99.4%, N=917, Deff=3.37, ICC=0.04, Figure 3B, Table S4). The *dhps* S436A mutation had an adjusted national prevalence of 30.8% (CI: 21.4–41.5%, N=917, Deff=8.2, ICC=0.13), occurring in a microhaplotype with *dhps* A437G in 290 of the 300 samples (96.7%) carrying it. The *dhps* I431V mutation was detected in four samples from Zaire province, with an adjusted national prevalence of 0.4% (CI 0.01–2.0%, N=917, Deff=2.6, ICC=0.03), always in a microhaplotype with *dhps* S436A and A437G. The *dhps* K540E mutation, the hallmark of the *dhps* double haplotype, had an adjusted national prevalence of 44.1% (CI 36.1–52.2%, N=872, Deff=4.38, ICC=0.06, Figure 3B). This mutation was less frequent in the northern provinces of Zaire and Uíge, where municipal allele frequencies were below 25%, while in southern Cuando Cubango, they exceeded 38% (Figure 3C, Table S5–7).

The SNPs of the *dhps* triple mutant haplotype, *dhps* A581G and A613S had a low prevalence, and were primarily found in the northern provinces (Figure 3B–C). The *dhps* A581G was detected in 14.2% of samples from Zaire, with an adjusted national prevalence of 3.1% (CI 0.9–7.7%, N=929, Deff=5.5, ICC=0.08) It was present in a microhaplotype with *dhps* K540E in 23 of the 27 samples (85.2%). *The dhps* A613S mutation was identified in four samples from Zaire in a haplotype with *dhps* K540E and A581G and in one sample from Namibe in a haplotype with *dhps* K540E and wild-type A581, with an adjusted national prevalence of 0.6% (CI 0.1–2.0%, N=914, Deff=2.0, ICC=0.02).

Mutations in *dhfr gene* had the following adjusted national prevalence: N51I 99.2% (CI: 97.9–99.8%, N=878, Deff=1.5, ICC=0.01), C59R 86.9% (CI: 80.3–91.9%, Deff=4.7, ICC=0.07), and S108N 99.8% (CI: 99.3–100.0%, N=916, Deff=0.76, ICC=0.00, Figure 3B). The C59R mutation was less frequent in Namibe and Benguela provinces, where municipal allele frequencies were ≤70% (Figure 3C, Table S5–7). The *dhfr* I164L mutation, associated with higher resistance levels, was not detected.

Participants older than over 24 years old had significantly higher odds of carrying the *dhps* K540E mutation (OR: 1.85; CI: 1.14–2.99; p=0.013, Table S9–10) compared to children under 5 years. Similarly, agricultural workers were significantly more likely to carry the *dhps* K540E mutation (OR: 1.95; CI: 1.11–3.45; p=0.021, Table S9,11) compared to those aged less than 5 and minors. Males were more likely to carry the *dhps* S436A mutation than females, with an odds ratio of 1.43 (CI: 1.05–1.94, p = 0.025). No significant associations were found between demographic factors and the presence of *dhps* A581G.

### Mutations in the k13 gene

**Figure 4.**
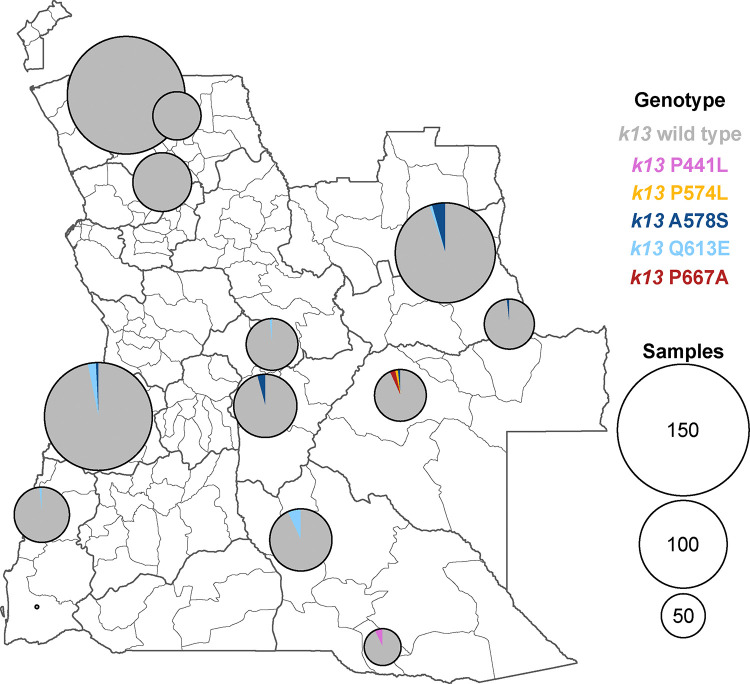
Non-synonymous mutations in *k13*. Proportion of samples carrying a nonsynonymous mutation in *k13* at the municipal level.

While a number of non-synonymous k13 mutations were detected, only two were found in >0.5% of samples (Table 2, Figure 4, Table S4–6): *k13* A578S was detected in Lunda Sul (4.1%, N=121), Bié (2.3%, N=132), Moxico (1.7%, N=116), and Benguela (0.8%, N=125), with an adjusted national prevalence of 1.0% (CI: 0.2–3.0%, N=931, Deff=2.6, ICC=0.03); and *k13* Q613E was detected in Cuando Cubango (4.4%, N=113), Benguela (2.4%, N=125), Namibe (1.5%, N=68), Lunda Sul (0.9%, N=115), and Bié (0.8%, N=132), with an adjusted national prevalence of 1.5% (CI: 0.3–4.4%, N=925, Deff=3.8, ICC=0.05).

Two markers of partial artemisinin resistance were observed: 1 (1.7%) sample from Luena municipality, Moxico province, carried the validated marker *k13* P574L, while 3 samples (7.3%) from Calai municipality, Cuando Cubango province, carried the candidate marker *k13* P441L. The *k13* P667A mutation was found in 2 (4.1%) samples in Luena municipality, Moxico province. Synonymous mutations were detected but at low proportions, including *k13* C494C, below 1%.

No significant association was found between the presence of non-synonymous *k13* mutations and age or gender (Table S8–9). No participant with a non-synonymous *k13* mutation reported traveling outside their province.

### Mutations in other genes

Nearly all samples, 748 out of 753 (99.3%), carried the wild-type *mdr1* N86 genotype. The adjusted national prevalence of *mdr1* N86Y was 0.63% (CI: 0.2–1.7%, Deff=1.07, ICC=0.00), with the highest proportion of samples in Zaire province, where 4.69% of samples carried the mutation (N = 128, Table S5–6). In contrast, *mdr1* Y184F had an adjusted national prevalence of 52.4% (CI: 47.90–56.85%, N = 901, Deff=1.42, ICC=0.01). Four samples, one each from Uíge, Cuando Cubango, Moxico, and Lunda Sul, carried *mdr1* D1246Y. No mutations were detected at positions 1034 or 1042. Among the 736 samples with complete genotypes at *mdr1* codons 86, 184 and 1246 whose haplotype could be inferred, 51.0% carried the NFD haplotype, and 73.8% carried the NYD haplotype (Table S15). No associations between the *mdr1* Y184F mutation and age, gender or occupation were observed (Table S9–11).

The PF3D7_1322700 T366I mutation had an adjusted national prevalence of 1.2% (CI: 0.3–3.1%, N = 760, Deff=1.91, ICC=0.02), with the highest prevalence in Benguela, where 4.9% (N = 103) of samples carried it (Table S5–6). Finally, the *mdr2* I492V mutation had an adjusted national prevalence of 61.8% (CI: 55.6–67.7%, N = 846, Deff=2.55, ICC=0.03) and was significantly less likely to be carried by participants aged 5–14 years compared to children under 5 years (OR: 0.65; CI: 0.43–0.96; p=0.031, Table S9–11).

## Discussion

In this study, we assessed the national prevalence of antimalarial drug resistance using samples collected across 8 of Angola’s 18 provinces using amplicon deep sequencing. Resistance makers prevalence was highly heterogeneous, with the *crt* K76T mutation, associated with chloroquine resistance, prevalent in the northwestern provinces (Uíge and Zaire), while *dhps* K540E, linked to SP resistance, most common in the eastern provinces. Although the *dhps* K540E allele was relatively rare in the northwestern provinces, the *dhps* A581G and *dhps* A613S mutations associated with higher levels of SP resistance were more common in these provinces. The validated ART-R *k13* P574L marker and the candidate *k13* P441L marker were detected at very low prevalence in only 2 southeastern provinces (Cuando Cubango and Moxico). The *mdr1* N86 wild-type genotype, associated with reduced lumefantrine susceptibility, was detected in nearly all samples analyzed.^30^ Low-level *P*. *falciparum* co-infections with *P*. *malariae*, *P*. *ovale* curtisi, and *P*. *ovale* wallikeri were identified.

Chloroquine was removed from Angola’s national malaria treatment guidelines in 2006 and replaced with three ACTs, artemether-lumefantrine, artesunate-amodiaquine, and dihydroartemisinin-piperaquine.^2^ The *crt* CVIET haplotype, observed at a national prevalence of 15.9%, is a strong predictor of chloroquine treatment failure but has a less clearly defined association with amodiaquine and quinine susceptibility.^26–28,48^ The *crt* CVIET haplotype and two additional *crt* mutations, *crt* A220S and *crt* I356T, which can confer even higher levels of chloroquine resistance in the presence of the *crt* CVIET haplotype, were detected at high frequencies in the northwestern provinces of Zaire and Uíge.^49^ These findings align with data from a 2018 study in Cabinda, a coastal exclave in northern Angola.^34^ Malaria molecular data from countries neighboring Angola are scarce, but two studies between 2013 and 2017 have shown that the CVIET haplotype, along with A220S and I356T mutations, are highly prevalent in regions of the DRC proximal to Zaire and Uíge provinces.^50,51^ Although chloroquine is no longer formally used for malaria treatment in Angola, the DRC, or other nearby countries, the results from this study suggest selective pressure for these mutations persists. Parasite migration across borders may also contribute to their continued presence in the region. The SVMNT haplotype, linked to amodiaquine resistance, detected in Angola’s capital Luanda in 2007, was not detected in this study.^52^

Mutations in the *dhps* and *dhfr* genes associated with SP in vitro and in vivo resistance impact treatment efficacy and may also reduce the efficacy of SP in chemoprevention.^31,53^ Like the *crt* mutations, the prevalence of the *dhps* and *dhfr* mutations was heterogeneous. The *dhps* K540E mutation, a hallmark of the quintuple mutant haplotype and a strong predictor of SP treatment failure in East and southern Africa was more prevalent in the eastern provinces, whereas sextuple mutant haplotypes were concentrated in the northwestern regions. The quintuple mutant haplotype has been linked to reduced SP efficacy for both treatment and perennial malaria chemoprevention in children.^53–56^ Additionally, seasonal malaria chemoprevention (SMC) has been shown to select for this haplotype.^57,58^

However, SP-based IPTp has been demonstrated to improve birth outcomes even in areas where the quintuple mutant haplotype is prevalent.^16–18^ Certain SNPs in the sextuple mutant haplotype, including the *dhps* A581G SNP - detected in 14.2% of samples from Zaire - have the potential to reduce the efficacy of SP for IPTp.^59–62^ Despite these concerns, the WHO continues to recommend SP for IPTp and SP in combination with amodiaquine for SMC, citing insufficient evidence of the negative impact of these mutations on intervention efficacy.^63^ Predominantly found in the northwestern province of Zaire in this study, the *dhps* A581G mutation was detected at very low levels in regions of the DRC neighboring Zaire in 2013–2014, and it reached a prevalence of nearly 10% in Cabinda in 2018 and at 3.4% in southern Republic of Congo in 2021.^34,50,64–66^ This suggests an increase over the last decade or a highly heterogeneous distribution in the region. In contrast, the *dhps* K540E mutation was more prevalent in eastern provinces. This mutation was also twice as likely to be carried by agricultural workers compared to minors, resembling associations previously observed in miners in the DRC.^64^ Overall, these findings highlight the geographical variation in *dhps* and *dhfr* mutations and the need for current information on mutation prevalence because of their potential impact on SP-based interventions targeting vulnerable populations.

The rapid rise of mutations in the k13 gene over the past decade has underlain the emergence of ART-R in Africa. The validated *k13* P574L marker, identified in a single sample from Moxico province, has also been detected at low frequencies in Western Zambia and Namibia’s Zambezi region, suggesting its localized regional circulation.^45,67^ This mutation has not been demonstrated to affect artemisinin efficacy in parasites with an African genetic background and appears to be rare across the rest of Africa. The candidate *k13* P441L marker, found in 3 samples from Cuando-Cubango, along the Namibia border, has been recently observed at a high prevalence in northern Namibia, southern and central Zambia, and southwestern Uganda.^23,45,67–69^ While robust clinical and laboratory evidence linking P441L to delayed clearance in Africa is lacking, a recent study in Myanmar reported a rise in its prevalence following a DHAP mass drug administration campaign.^70^

Other non-validated mutations previously detected in the region were also identified: the *k13* P667A mutation recently reported at high prevalence in eastern Zambia, was found in 2 samples from Moxico; mirroring the geographical patterns observed for *k13* P574L. Limited laboratory data suggest that *k13* P667T/S mutations are associated with delayed clearance, pointing to a possible phenotype for *k13* P667A. The *k13* Q613E mutation, detected at low prevalence in 5 provinces, has been reported at low prevalence in Zambia, Angola and the DRC.^36,71–73^ This mutation had no effect in vitro clearance of a single laboratory isolate.^71^ Similarly, *k13* A578S, found at low prevalence in 4 provinces, has been documented across Africa without evidence of impacting artemisinin susceptibility.^74^

The clinical impact of k13 validated and candidate markers, as well as other novel mutations, on the efficacy of ACT and artemisinin monotherapy in Angola remains to be studied. However, their low prevalence suggests that they are not an immediate concern. ACT efficacy has been monitored every two years in Angola since 2013, with AL efficacy after molecular correction falling below 90% in four of the five surveys conducted in Zaire and in the 2019 survey in Lunda Sul.^11–15^ This study did not find any *k13* markers of ART-R or mutations in the coronin gene previously linked to artemisinin resistance.^75^ These findings are consistent with the high TES day-3 clearance rates, which suggest no clinical evidence of ART-R.

Although reduced lumefantrine susceptibility has been reported in Uganda, no validated molecular marker for lumefantrine resistance exists, and there have been no reports suggesting clinically significant changes in its efficacy.^3,76,77^ The *mdr1* N86 genotype, associated with lower lumefantrine susceptibility, was found in almost all samples across all provinces and is unlikely to explain the potential reduction in AL efficacy in Zaire.^30^ While AL efficacy in Zaire has remained below 90%, it has been relatively stable since 2013.^11–15^ Furthermore, molecular correction methods used in TES have inherent technical limitations that could introduce biases into these estimates.^78,79^ At the time of writing, a new study reported an association between increased *mdr1* copy number and reduced AL treatment efficacy in Bengo province in northern Angola.^80^ This study did not assess *mdr1* CNV. Together, these data suggest that Angola may be facing emerging ACT efficacy challenges on two fronts: (1) reduced clinical efficacy of AL in the northwest and (2) the circulation of *k13* mutations in the southeast, which, though currently rare, could increase given their rising prevalence in neighboring countries.^45,67^ Angola currently recommends three ACTs - AL, ASAQ and DP - for malaria treatment, without systematic rotation or predefined geographic targeting. In line with evolving strategies in Eastern Africa and current WHO recommendations, the Angolan National Malaria Control Program is actively discussing the adoption of rotational, geography-based multiple first-line therapies to prevent and contain the spread of ACT resistance.^81^ Encouragingly, the efficacy of non-AL ACTs in the country remains very high.^11–15^

This study had several limitations. Samples were collected during a single season, restricting the assessment of temporal trends. The sampling strategy aimed to estimate country-level prevalence with broad geographic coverage but was not designed to detect fine-scale spatial patterns and did not cover all provinces, leaving vast geographical areas unsurveyed. Delays in study initiation resulted in some health facilities in low transmission strata not achieving the required sample size. While the majority of health facilities were randomly selected, TES sentinel sites were deliberately included, which may have violated the statistical assumptions underlying the use of survey weights to correct for sampling bias. Additionally, the low prevalence of most mutations of interest and missing data for certain demographic variables, including all the demographic data from the Lunda Sul province, limited the sample size available for analyzing associations between mutations and demographic factors. Allele frequencies were estimated only at the municipal level, as the assumption of panmixia is unlikely to hold at larger geographic scales. Technical limitations of the molecular assay included difficulties in phasing SNPs from separate amplicons for haplotype reconstruction.

This study provides an up-to-date overview of the distribution of mutations associated with antimalarial resistance in Angola, revealing marked geographic heterogeneity: higher prevalence of SP resistance markers in the east, low levels presence of ART-R markers in the southeast, and high frequencies of markers linked to chloroquine and higher-level SP resistance in the northwest. While the findings do not indicate an expansion of known ART-R markers that could compromise ACT efficacy, they highlight the persistent risk of selection and spread of resistance. Together with evidence of reduced efficacy from TES, these results reinforce the urgent need for continued surveillance. This study also underscores the value of molecular approaches as a powerful means to generate actionable data. Angola must remain prepared to monitor and respond to changes in parasite populations and to develop proactive strategies adapted to the local context to prevent and contain emerging threats. Leveraging insights from studies like this one will be essential to protect treatment efficacy and sustain progress toward malaria elimination.

## Supplementary Material

Supplement 1

## Figures and Tables

**Table 1.** Demographics of study participants with successfully sequenced samples.

Characteristic	Overall	Benguela	Bié	Cuando Cubango	Moxico	Namibe	Uíge	Zaire
**N**	722 (817*)	113	118	93	105	57	105	131
**Health Facilities**	14 (16*)	2	2	2	2	2	2	2
**Age**								
**< 5 years**	168 (23%)	23 (20%)	37 (31%)	18 (19%)	33 (31%)	9 (16%)	20 (19%)	28 (21%)
**5 – 14 years**	287 (40%)	68 (60%)	43 (36%)	35 (38%)	33 (31%)	22 (39%)	33 (31%)	53 (40%)
**15 – 24 years**	156 (22%)	11 (9.7%)	12 (10%)	30 (32%)	23 (22%)	19 (33%)	31 (30%)	30 (23%)
**>25 years**	111 (15%)	11 (9.7%)	26 (22%)	10 (11%)	16 (15%)	7 (12%)	21 (20%)	20 (15%)
**Gender**								
**Female**	384 (53%)	63 (56%)	66 (56%)	43 (46%)	63 (60%)	26 (46%)	62 (59%)	61 (47%)
**Male**	338 (47%)	50 (44%)	52 (44%)	50 (54%)	42 (40%)	31 (54%)	43 (41%)	70 (53%)
**Occupation**								
**Agriculture**	66 (9.2%)	0 (0%)	17 (14%)	8 (8.6%)	7 (6.7%)	0 (0%)	28 (27%)	6 (4.6%)
**Student**	297 (41%)	55 (49%)	27 (23%)	38 (41%)	38 (36%)	27 (50%)	35 (34%)	77 (59%)
**Other**	68 (9.5%)	11 (9.7%)	12 (10%)	7 (7.5%)	11 (10%)	13 (24%)	5 (4.8%)	9 (6.9%)
**Minor**	257 (36%)	44 (39%)	57 (48%)	31 (33%)	43 (41%)	11 (20%)	35 (34%)	36 (27%)
**Unemployed**	30 (4.2%)	3 (2.7%)	5 (4.2%)	9 (9.7%)	6 (5.7%)	3 (5.6%)	1 (1.0%)	3 (2.3%)
**Unknown**	4	0	0	0	0	3	1	0
**Travel history**								
**Domestic**	49 (6.8%)	7 (6.2%)	16 (14%)	19 (20%)	6 (5.7%)	0 (0%)	1 (1.0%)	0 (0%)
**International**	1 (0.1%)	0 (0%)	0 (0%)	0 (0%)	0 (0%)	1 (1.8%)	0 (0%)	0 (0%)
**No travel reported**	672 (93%)	106 (94%)	102 (86%)	74 (80%)	99 (94%)	56 (98%)	104 (99%)	131 (100%)

*: Includes 95 participants from Lunda Sul province, for which demographic data was unavailable.

**Table 2.** Summary of non-synonymous mutations. Markers of partial artemisinin resistance (ART-R) are categorized as validated or candidate, following the current classification of the World Health Organization.^47^

Gene	Observed in >0,5% of samples	Observed in <0,5% of samples
***k13***	No evidence of ART-R A578S, Q613E	Validated ART-R markers P574L Candidate ART-R markers P441L No evidence of ART-R P667A
***crt***	M74I, N75E, K76T, A220S, I356T	F48L
***dhfr***	N51I, C59R, S108N	
***dhps***	S436A, A437G, K540E, A581G, A613S	I431V
***mdr1***	N86Y, F184Y	D1246Y
***mdr2***	I492V	
**PF3D7_1322700**	T236I	
